# Aspects épidémiologique, clinique, thérapeutique et évolutif de la maladie de Basedow en Médecine Interne au CHU Ledantec Dakar (Sénégal)

**DOI:** 10.11604/pamj.2016.25.6.7868

**Published:** 2016-09-14

**Authors:** Nafissatou Diagne, Atoumane Faye, Awa Cheikh Ndao, Boundia Djiba, Baidy Sy Kane, Souhaibou Ndongo, Abdoulaye Pouye

**Affiliations:** 1 Médecine Interne, Centre Hospitalier Universitaire Aristide Le Dantec, Dakar, Sénégal

**Keywords:** Auto-Immunité, hyperthyroïdie, Dakar, Autoimmunity, hyperthyroidism, Dakar

## Abstract

La maladie de Basedow est une affection auto immune associant une thyrotoxicose à des manifestations de fréquence variable comme le goitre, l'ophtalmopathie et le myxœdème prétibial. Son diagnostic est souvent facile, tandis que sa prise en charge demeure encore difficile. L'instauration d'un traitement médical simple expose à un risque de récidive. Au Sénégal et en Afrique Sub-saharienne peu d'études ont porté sur la maladie de Basedow. L'objectif de l'étude était de décrire les aspects épidémiologique, clinique, thérapeutique et évolutif de la maladie de Basedow en milieu hospitalier à Dakar. Il s'agissait d'une étude rétrospective menée du 1er janvier 2010 au 31 décembre 2013 dans le service de Médecine Interne du centre hospitalier universitaire Aristide Le Dantec. Durant la période, 108 patients suivis en consultation externe pour maladie de Basedow, ont été inclus sur un total de 834 patients suivis en consultation externe. le diagnostic a été retenu devant les signes cliniques, biologiques et immunologiques. Cent huit patients, atteints de maladie de Basedow ont été inclus sur un total de 834 consultations. Le sex ratio était de 7,3 et la moyenne d'âge de 34,6 ans. Les principaux motifs de consultation étaient : les palpitations et l'amaigrissement dans respectivement 46,3% et 39,8% des cas. Le syndrome de thyrotoxicose était présent chez 93,5% des patients, un goitre était noté chez 87% des patients et une exophtalmie chez 78,7% des patients. La principale complication était à type de cardiothyréose retrouvée chez 11,1% des patients. Tous les patients ont eu un traitement par antithyroïdiens de synthèse. L'évolution a été favorable dans 19,4% des cas. Une récidive à été notée dans 57% des cas et dans 23,1% des cas les patients ont été perdus de vue. La maladie de Basedow est la cause de la plus fréquente d'hyperthyroïdie. Le tableau est dominé par les manifestations cliniques liées à l'hyper métabolisme de l'organisme. Dans cette étude, il apparait que la thyroïdectomie n'est pas de première intention si l'on connait le nombre élevé de récidive après le traitement médical.

## Abstract (en)

Basedow-Graves disease is an autoimmune affection characterized by the association of thyrotoxicosis with variable frequency events such as goiter, ophthalmopathy and pretibial myxedema. Its diagnosis is often easy, while its management remains difficult. A simple medical treatment exposes patient to recurrence risk. In Senegal and Sub-Saharan Africa few studies have focused on Basedow-Graves disease. This study aims to describe the epidemiological, clinical, therapeutic and evolutionary aspects of Basedow-Graves disease at a Hospital in Dakar. This was a retrospective study conducted from 1 January 2010 to 31 December 2013 in the Department of Internal Medicine at the Aristide Le Dantec University Hospital. During this period, 108 patients receiving outpatient treatment for Basedow-Graves disease were included out of a total of 834 patients receiving outpatient treatment. The diagnosis was made on the basis of clinical, biological and immunological signs. One hundred and eight patients suffering from Basedow-Graves disease were included out of a total of 834 consultations. Sex ratio was 7.3 and the average age was 34.6 years. The main reasons for consultation were: palpitations and weight loss in 46.3% and 39.8% of cases respectively. Thyrotoxicosis syndrome was found in 93.5% of patients, goiter was found in 87% of patients and exophthalmos in 78.7% of patients. The main complication was cardiothyreosis found in 11.1% of patients. All patients underwent antithyroid synthetic drugs treatment. The evolution was favorable in 19,4% of cases. Disease recurrence was observed in 57% of cases and in 23.1% of patients were lost to follow-up. Basedow-Graves disease is the most common cause of hyperthyroidism, The patient’s clinical picture is dominated by manifestations related to hypermetabolism. This study highlights that thyroidectomy isn’t the first-line of treatment if we consider the high number of recurrences after medical treatment.

## Introduction

Les hyperthyroïdies sont parmi les affections endocriniennes les plus fréquentes. La principale étiologie retrouvée est la maladie de Basedow. Le diagnostic de la maladie de Basedow est souvent simple La maladie de Basedow est une maladie auto-immune de la thyroïde qui se manifeste par une hyperthyroïdie, un goitre homogène et parfois une ophtalmopathie [1]. La maladie de Basedow correspond à 70% des hyperthyroïdies en France [2]. En Afrique des pourcentages similaires sont retrouvés, au Cameroun il s'agit de 82% des hyperthyroïdies [3]. Au Sénégal il n'existe pratiquement pas de données sur la prévalence de la maladie de Basedow. Le traitement permet d'éviter les nombreuses complications, notamment cardio-vasculaires. Mais il reste néanmoins difficile et se base sur un arsenal thérapeutique comprenant les antithyroïdiens de synthèse, l'iode radioactif et la chirurgie [1]. Au Sénégal, les études sont rares sur la prévalence de la maladie de Basedow. Ainsi, les objectifs de ce travail étaient de décrire les aspects épidémiologique, clinique, thérapeutique et évolutifs des patients porteurs de la maladie de Basedow suivis en ambulatoire dans le service de médecine interne du CHU Aristide Le Dantec.

## Méthodes

Il s'agissait d'une étude rétrospective descriptive étude rétrospective portant sur des patients atteints de la maladie de Basedow suivis dans le service de médecine interne de l'hôpital Aristide Le Dantec. On été inclus tous les dossiers des malades suivis en ambulatoire dans le service pour une maladie de Basedow durant la période du 1^er^Janvier 2010 au 31 décembre 2013. Le diagnostic de maladie de Basedow à été retenu devant les critères suivants: un syndrome de thyrotoxicose clinique, avec au minimum une diminution du taux de TSH ou une augmentation de la T_4_ ou de la T_3_; ces deux critères devaient être associés obligatoirement à une exophtalmie et/ou un taux d'anticorps anti-récepteur de la TSH positif.

Ont été exclus tous les malades qui ne présentaient pas à la clinique une exophtalmie ou à la biologie un taux positif d'anticorps anti-RTSH. Les paramètres étudiés étaient les données épidémiologiques, cliniques, paracliniques, thérapeutiques et évolutives. Un dépouillement de tous les dossiers des malades ayant consulté pour maladie de Basedow dans le service de médecine interne à été réalise. Le recueil des données a été effectué à l'aide de fiches pré imprimées. Les informations recueillies ont été étudiées à l'aide du logiciel épi-info version 3.5.4 Pour les différentes variables, nous avons fait une analyse descriptive.

## Résultats

De Janvier 2010 à Décembre 2013, 834 patients ont consulté dans le service de médecine interne. Cent cinquante patients, soit 18% avaient une hyperthyroïdie. La maladie de Basedow en était la principale cause, et concernait 108 patients soit 72% des cas d'hyperthyroïdie. Les patients de sexe féminin représentaient 88% des cas. La moyenne d'âge était de 34,6 ans, avec des extrêmes allant de 13 à 69 ans. La majorité des patients (80,6%) provenait de la ville de Dakar. En moyenne les patients consultaient 12,3 mois après le début des symptômes, avec des extrêmes allant de 5 à 26 mois. La notion d'épine irritative a été recherchée chez 67 patients, soit dans 62% des cas. Parmi eux, 46 (42,6%) ont rapporté une épine irritative. Les conflits familiaux étaient au premier plan notés, dans 45,6% des cas.

Les signes fonctionnels les plus fréquemment retrouvés étaient les palpitations et l'amaigrissement dans respectivement 46,3% et 39,8% des cas (voir Tableau 1). Cent un patients ont présenté des signes de thyrotoxicose, soit 93,5%. Les principaux signes étaient dominés par les signes cardiovasculaires retrouvés chez 79,6%des patients (Tableau 2). Le goitre était retrouvé chez 94 patients, soit 87% des cas. Le caractère vasculaire à la clinique n'a été retrouvé que dans 45,7% des cas. Les manifestations oculaires étaient présentes chez 102 patients, soit 94,4% des cas. Une exophtalmie était notée chez 78,7% des patients. Le myxœdème pré-tibial n'a été observé que chez 3 patients soit 2,8% des cas. Trois cas de pathologies auto-immunes associées à la maladie de Basedow ont été observées dans cette étude soit 2,8%. Il s'agissait de deux cas polyarthrite rhumatoïde, un cas de lupus érythémateux systémique. Des antécédents familiaux de pathologies auto-immunes ont été retrouvés chez 13 patients (12%). Il s'agissait de la maladie de Basedow chez 12 d'entre eux (11,1%) et d'un cas de polyarthrite rhumatoïde. Les complications ont été constatées chez 13 patients, soit 12%. La cardiothyréose a été la plus fréquemment retrouvée, chez 12 patients (11,1%). Seul un cas d'orbitopathie maligne a été rapporté, soit 0,9% des cas de complications. Aucun cas de crise aigüe thyrotoxique, n'a été retrouvée dans l'étude. La TSH était basse chez tous les patients, le taux moyen de était de 0,007 µUI/ml avec des extrêmes allant de 0,001 à 0,4 µUI/ml. Le dosage des anticorps anti-récepteurs de la TSH a été réalisé chez 28 patients (25,9%). Le taux moyen était de 26,8 UI/l avec des extrêmes allant de 1,8 à 124,8 UI/l. Il était positif chez tous les patients. L'échographie était réalisée chez 44 patients (40,7%). Le caractère hyper vasculaire a été observé chez la majorité des patients (86,4%).

**Tableau 1:** Principaux motifs de consultation

Principal motif	Fréquence	Pourcentage
Palpitations	50	46,3%
Amaigrissement	43	39,8%
Orbitopathie	41	38%
Tremblements	36	33,3%
Goitre	35	32,4%
Nervosité	26	24,1%
Insomnie	22	20,4%
Thermophobie	21	19,4%
Dyspnée	16	14,8%
Cardiothyréose	4	3,7%

**Tableau 2:** Signes de thyrotoxicose

Thyrotoxicose	Fréquence	Pourcentage
Troubles cardiovasculaires	86	79,6%
Troubles neuropsychiques	62	57,4%
Amaigrissement	43	39,8%
Thermophobie	21	19,4%
Asthénie	15	17,6%
Diarrhée	6	5,5%
Polyphagie	4	3,7%
Amyotrophie	2	1,9%
Polyurie	2	1,9%
Polydipsie	1	0,9%

Les bétabloquants ont été prescrits chez 82 patients (75,9%) et les anxiolytiques chez 40,7%. Tous les patients ont bénéficié d'un traitement par antithyroïdiens de synthèse. Dans notre étude, tous les patients ont bénéficié d'un traitement par antithyroïdiens de synthèse. Seuls 3 patients ont bénéficié d'une chirurgie thyroïdienne, soit 2,8%. Il s'agissait d'une thyroïdectomie subtotale dans les 3 cas. L'évolution était favorable sur le plan clinico-biologique chez 21 patients (19,4%). Leurs hormones thyroïdiennes ont été normalisées après un traitement bien conduit d'au moins 18 mois. Par ailleurs, 62 patients (57,4%) ont évolué défavorablement. Il s'agissait d'une récidive de l'hyperthyroïdie chez 25 d'entre eux (23,1%) et d'un échec chez 37 soit 34,3%, et dans 23,1% des cas, les patients ont été perdus de vue.

## Discussion

Parmi 150 patients reçus pour hyperthyroïdie, 108 cas étaient liés à la maladie de Basedow, soit une prévalence de 72%. Ces mêmes données ont été retrouvées en Europe. En France, les hyperthyroïdies sont liées à la maladie de Basedow dans 70% des cas [2]. Au Congo, Mbadinga [4] dans une étude rétrospective portant sur 72 cas d'hyperthyroïdie, avait noté 72,2% de cas de maladie de Basedow. Plusieurs études ont montré la nette prédominance féminine observée dans la maladie de Basedow avec des prévalences de 19 pour 1000 femmes contre 1,6 pour 1000 hommes [5]. Cette prédominance féminine se retrouvait dans cette étude avec un sex-ratio de 7,3. Des résultats similaires ont été notés au Congo par Mbadinga [4] ou sex-ratio était de 7,7 Lokrou [6] un sex-ratio de 8. La moyenne d'âge dans cette étude était de 34,6 ans avec des extrêmes allant de 13 à 69 ans. En France, Bilosi [7] dans son étude concernant 128 patients, retrouvait des résultats similaires avec une moyenne d'âge de 34 ans.

Dans son étude, Delevaux [8] affirme que le stress peut être un élément déclencheur de thyroïdite auto-immune et qu'ainsi, les patients porteurs d'une maladie de Basedow rapportent plus souvent que la population témoin, un événement traumatisant. Parmi les 67 patients chez qui une épine irritative a été recherchée, 46 (42,6%) avaient rapporté un facteur déclenchant de leur maladie. Ould Isselmou [9], avait objectivé que le facteur déclenchant le plus incriminé était le divorce. Sur le plan clinique 101 patients (93,5%) avaient présenté un syndrome de thyrotoxicose. Les signes les plus fréquemment retrouvés étaient : Les signes cardiovasculaires dans 79,6% des cas, l'amaigrissement avec une fréquence de 39,8%, les tremblements dans 33,3% des cas. Dans l'étude de Lokrou [10] la tachycardie était le symptôme le plus fréquent (82% des cas). Les tremblements et l'amaigrissement étaient observés à des fréquences respectives de 72% et 66%. En Algérie [11], il s'agissait principalement des palpitations et de l'amaigrissement dans 59% et 41% des cas. Nos fréquences beaucoup plus basses que dans les autres études pourraient s'expliquer par le fait que dans nos dossiers les signes cliniques du syndrome de thyrotoxicose n'étaient pas toujours détaillés dans les observations.

Dans la population mondiale, la prévalence du goitre est importante, estimée à 15,8% [12]. Le goitre était fréquemment retrouvé dans cette étude chez 94 patients, soit 87% des cas. Le caractère vasculaire dans 45,7% des cas. Dans les pays occidentaux, notamment en France, les goitres étaient estimés à 80,8% et aux Etats-Unis, Larsen [13] retrouvait une fréquence de 100%. Lokrou (10) retrouvait un goitre vasculaire dans 56% des cas. L'orbitopathie avait été notée chez 94,4% des patients, et l'exophtalmie chez 78,7% d'entre eux. Dans les pays occidentaux, Morax [14] estimait l'orbitopathie basedowienne entre 20 et 40% selon la prise en compte ou non des signes palpébraux. Il estimait l'exophtalmie à 63%. Lokrou [10] estimait sa fréquence à 70%. En Algérie [1] et Au Maroc [15], l'exophtalmie avait une fréquence plus faible 17,5%. Il en ressort que l'exophtalmie est plus fréquente chez les sujets de race noire. En effet, il existe des variations connues des valeurs de l'exophtalmie d'une race à l'autre. Elle est moins marquée chez les asiatiques que chez les caucasiens alors qu'elle l'est plus chez les patients de race noire ayant des orbites plus étroites [14]. Dans cette étude 3 patients avaient un myxœdème pré-tibial, soit 2,8%. Aux Etats-Unis [13] il est estimé à 5%.

L'association d'autres maladies auto-immunes au cours des dysthyroïdies peut être expliquée par des mécanismes physiopathologiques communs et justifient la recherche complète des autres pathologies auto-immunes [16]. Dans cette étude, seuls 2,8% des patients ont présenté une autre pathologie auto-immune associée à leur maladie de Basedow. En Tunisie [17], le lupus et le syndrome des anti phospholipides étaient les maladies auto-immunes les plus fréquemment associées à la maladie de Basedow avec une prévalence de 28,6% des cas. Puis étaient retrouvés le syndrome de Gougerot-Sjogren et l'anémie de Biermer dans 14,3% des cas. Dans la littérature, la fréquence de la cardiothyréose varie de 10 à 60% [18], ceci en fonction du mode de recrutement endocrinologique ou cardiologique. La cardiothyréose a été notée chez 11,1% des patients. En effet, pour Lokrou [10], 14% des patients atteints de la maladie de Basedow ont présenté une cardiothyréose. Cette fréquence faible de la cardiothyréose pourrait s'expliquer par le mode de recrutement endocrinologique dans cette étude.

Dans cette étude, le dosage des anticorps n'était pas réalisé systématiquement. Les anticorps anti RTSH avaient été réalisés chez 28 patients et étaient positifs chez 100% d'entre eux. Une étude réalisée au Maroc [15], retrouve pour les anticorps anti RTSH à un taux positif dans 87,5% des cas. La demande non systématique des anticorps pourrait s'expliquer dans un premier temps par la grande fréquence de l'exophtalmie qui est un signe spécifique de la maladie de Basedow, mais également du fait du contexte économique qui limitait la demande de ces examens immunologiques dans notre étude. L'échographie thyroïdienne a été réalisée chez 44 des patients (40,7%). Parmi ces goitres, 86,4% ont présenté un aspect hyper vasculaire. Une étude réalisée par Anadam [15] avait objectivé un goitre hyper vasculaire dans 82,5% des cas à l'échographie. Les médicaments antithyroïdiens ont pour effet de restaurer l'euthyroïdie le plus rapidement possible. Ils ne « guérissent » pas au sens propre la maladie, mais en réduisant la synthèse des hormones, peuvent permettre d'attendre, peut être même de favoriser la rémission spontanée de la maladie [1]. Dans l'étude, le traitement de référence était le traitement médical par antithyroïdiens de synthèse. Les hormones thyroïdiennes ont été prescrites chez 17,6% des patients et seulement en cas d'hypothyroïdie iatrogène. Dans l'étude de Ghissani [19], le traitement médical exclusif a été prescrit dans 42% des cas Dans cette étude, le traitement radical était à type de thyroïdectomie subtotale et n'a été réalisée que chez 3 patients. Au Maroc [19], le traitement radical en 2ème intention concernait 58% des cas, dont 19% de thyroïdectomie.

Dans la littérature, le taux de récidive après traitement médical par antithyroïdiens de synthèse atteint 50%. Afin d'avoir un effet optimal des ATS, la durée du traitement doit être comprise entre 12 et 24 mois [1]. Dans cette étude, l'évolution était favorable chez 19,4% des patients et était marquée par une normalisation des hormones thyroïdiennes après traitement médical bien suivi pendant une période de 18 mois. Chez 62 patients elle était défavorable, marquée soit par une récidive dans 23,1% des cas, soit un échec du traitement médical dans 34,3% des cas. Une étude prospective publiée par Allanic [20], comparant les effets respectifs d'un traitement de 6 mois et de 18 mois, montre que 58,3% des patients traités pendant 6 mois rechutent. Ce chiffre diminue à 38,2% chez les patients traités pendant 18 mois. Il faut noter que dans cette étude, la surveillance du traitement médical était relativement courte, de 24 à 30 mois tout au plus après normalisation des hormones. Donc l'évolution après cette période reste inconnue.

## Conclusion

La maladie de Basedow demeure la principale étiologie des hyperthyroïdies. Certains signes sont quasi constants comme les signes cardiovasculaires et le goitre. Dans cette étude l'exophtalmie est fréquente dans la maladie de Basedow. La cardiothyréose est la principale complication pouvant engager le pronostic vital. Le traitement médical peut être efficace mais seulement dans la moitié des cas. Une récidive est très fréquente après traitement médical. Après échec thérapeutique la seule alternative est le traitement radical comme la thyroïdectomie qui est rarement pratiquée en Afrique.

### Etat des connaissances actuelle sur le sujet

- La maladie de Basedow est la principale cause d'hyperthyroïdie;
- C'est une maladie de la femme jeune le sexe ratio est en général de 7 en faveur des femmes;
- Le traitement en première intention fait appel aux antithyroïdiens de synthèse.

### Contribution de notre étude à la connaissance

- C'est une étude qui est rarement publié en Afrique sub-saharienne et au Sénégal;
- L'orbitopathie basedowienne est retrouvée en général entre 20 à 40% chez les patients, alors que dans notre étude elle a été notée chez 94,4% des patients et l'exophtalmie dans 78,7% des cas;
- Cette étude nous a permis de voir que le traitement radical tel que la chirurgie est rarement pratiquée alors que l'évolution était défavorable dans57,4% des cas avec une récidive chez 23,1% des patients et un échec chez 34,3% des patients.
